# Characterization and Mechanistic Study of Heavy Metal Adsorption by Facile Synthesized Magnetic Xanthate-Modified Chitosan/Polyacrylic Acid Hydrogels

**DOI:** 10.3390/ijerph191711123

**Published:** 2022-09-05

**Authors:** Liming Dong, Chengyang Shan, Yuan Liu, Hua Sun, Bing Yao, Guizhen Gong, Xiaodong Jin, Shifan Wang

**Affiliations:** 1 School of Material and Chemistry Engineering, Xuzhou University of Technology, Xuzhou 221018, China; 2 Department of Forensic Science and Technology, Jiangsu Police Institute, Nanjing 210031, China

**Keywords:** magnetic hydrogels, chitosan, adsorption mechanism, heavy metal, wastewater treatment

## Abstract

A simple method was used to synthesize magnetic xanthate-modified chitosan/polyacrylic acid hydrogels that were used to remove heavy metal ions from an aqueous solution. Xanthate modification of chitosan significantly improved adsorption performance: individual adsorption capacities of the hydrogel for Cu(II), Cd(II), Pb(II), and Co(II) ions were 206, 178, 168, and 140 mg g^−1^, respectively. The magnetic hydrogels had good regeneration ability and were effectively separated from the solution by use of a magnet. Adsorption kinetic data showed that the removal mechanism of heavy metal ions from the solution by magnetic hydrogels occurs mainly by chemical adsorption. The equilibrium adsorption isotherms were well-described by the Freundlich and Langmuir equations. Positive values were found for the Gibbs standard free energy and enthalpy, indicating an increase in the disorder at the solid–liquid interface during adsorption. Magnetic xanthate-modified chitosan-based hydrogels that exhibit high adsorption efficiency, regeneration, and easy separation from a solution have broad development prospects in various industrial sewage and wastewater treatment fields.

## 1. Introduction

Magnetic hydrogels are a class of environment-sensitive hydrogels that are responsive to magnetic fields. Their movement mode and direction can be rapidly and effectively controlled by application of an external magnetic field. By incorporating magnetic particles into different hydrogel matrix materials, various products with useful properties and functions can be obtained. Examples include adsorption-like magnetic hydrogels that can effectively remove harmful substances from water [1] and magnetic hydrogels that can target and release drugs to achieve high-efficiency therapeutic effects [2]. Some magnetic hydrogel materials with good biocompatibility are widely used in tissue engineering and other fields [3].

A magnetic hydrogel adsorbent is composed of a carrier magnet and a matrix. Ordinary carrier magnets include zero-valent iron nanoparticles, magnetite (Fe_3_O_4_), hematite (α-Fe_2_O_3_), maghemite (γ-Fe_2_O_3_), and spinel ferrite; the matrix can include carbon, polymer, starch, or biomass, amongst others [4,5]. The adsorption mechanism and kinetic process depend on the surface morphology and magnetic properties of the adsorbent, as well as adsorption conditions, such as pH, time, adsorbent concentration, wastewater temperature, and initial dose of pollutant [6]. Current research on magnet carriers is focused on the use of Fe_3_O_4_ particles: nanoscale particles, in particular, are very effective for removing metal ions [7].

Preparation methods for adsorbents mainly include co-precipitation, in situ synthesis, and grafting cross-linking [2]. The latter, wherein the grafting and cross-linking processes combine the nanoparticles and hydrogel network into an overall stable cross-linked network, is considered to be the best preparation route; however, it is limited by the number of active functional groups on the Fe_3_O_4_ particle surfaces, resulting in poor cyclic stability of the adsorbent, which is the main obstacle to commercialization of this method [8]. 

Chitosan is an excellent candidate for adsorption of heavy metal ions from aqueous solutions because of its multiple chelation sites, as well as amino and hydroxyl groups that attract metal ions through coordination bonds or ion exchange [9,10]. Many studies have focused on evaluating the adsorption properties of chitosan and its modified forms for removal of various heavy metals [11,12]. A phosphorylated chitosan-coated magnetic Fe_3_O_4_@SiO_2_ nanoparticle hydrogel bead (Fe_3_O_4_@SiO_2_@CS-P) exhibited highly selective adsorption of Pb(II), with an adsorption capacity of 212.8 mg/g. Competitive experiments were conducted in a solution containing seven metal ions, and Fe_3_O_4_@SiO_2_@CS-P exhibited excellent Pb(II) capture selectivity, with a distribution coefficient (0.75 L g^−1^) more than ten times that of other metal ions [13]. Post-adsorption Fourier-transform infrared spectroscopy and photoelectron spectroscopy indicated that the high adsorption performance and selective capture of Pb(II) were mainly controlled by coordination of the phosphate groups on the adsorbent surface. Thiol-based modified magnetic hydrogels were synthesized using polyvinyl alcohol, chitosan, and magnetic Fe_3_O_4_@SiO_2_ nanoparticles, and gave efficient removal of heavy metal ions from aqueous solutions. These hydrogels exhibited a removal capacity of 307 mg g^−1^ for Cd(II), had good regeneration ability, and were effectively separated from the solution by application of a magnetic field [14].

To improve the adsorption of magnetic chitosan materials for application to wastewater and selective-ion adsorption, most research concerns mixtures of new materials and modification of well-known materials [15]. However, comprehensive evaluation of the stability of magnetic chitosan in the adsorption process is relatively lacking, and research on recovery and regeneration performance is even more insufficient [16]. In this study, the stability of a modified magnetic chitosan material was analyzed under different conditions, appropriate to practical applications, by establishing a thermodynamic–kinetic model. Attaching the modifier and magnetic components to the chitosan material avoids the phenomenon that the heavy metal adsorption process only involves the transfer of ions, and the adsorbed metal ions can be recovered and reused. This approach enables fundamental control of the problem of wastewater treatment [17,18]. To ensure good magnetic properties of the absorbent, Fe_3_O_4_ wrapped in SiO_2_ was used as the magnetic core. Its magnetic characteristics were used to facilitate recycling, which can save costs and avoid economic losses and secondary environmental pollution [19].

In this study, we designed and synthesized a novel magnetic xanthate-modified chitosan/polyacrylic acid (MXCS/PAA) hydrogel to improve the adsorption capacity, reproducibility, and stability of chitosan-based magnetic hydrogels. PAA could be grafted onto CS by via situ polymerization. Meanwhile, the strong electrostatic interaction between the carboxyl group of PAA and the amino group of CS could promote the formation of composite particles under very mild conditions without surfactants. Modifying chitosan with xanthate can improve the interactions between the chitosan and heavy metals in solution, which will increase the adsorption capacity. The synthesized hydrogel was characterized, and its adsorption properties for the removal of Cu(II), Cd(II), Pb(II), and Co(II) from the aqueous solution studied. Adsorption kinetics and isotherms were determined, which are essential for applying these materials for heavy metal removal.

## 2. Materials and Methods

### 2.1. Materials

Chitosan, AA were purchased from Sinopharm Chemical Reagent Co., Ltd. (Shanghai, China), other reagents and chemicals were purchased from Aldrich (St. Louis, MO, USA), Acros (Geel, Belgium) and Fisher Scientific (Hampton, NH, USA) unless otherwise noted and used without further purification. All other chemicals were commercially available analytical-grade reagents. All solutions were prepared with deionized water.

### 2.2. Preparation of MXCS/PAA

First, sodium polyacrylate was prepared, 2.2 mL of acrylic acid solution was added to 5 mL of deionized water and then 3.2 mL of 40% NaOH solution was added, stirring evenly to make the acrylic acid completely mixed to obtain sodium polyacrylate. Then, 3 g of chitosan was placed into a three-necked flask and 2% acetic acid solution was added and mechanically stirred to dissolve it. After being completely dissolved, the previously prepared sodium acrylate was added, then the initiator 0.08 g K_2_S_2_O_8_ (KPS) was added, followed by Fe_3_O_4_@SiO_2_ (1.2 g) into the mixture. Subsequently, the composite gel-forming solution was stirred continuously for 3 h at 30 °C until the Fe_3_O_4_@SiO_2_/CS/PAA (MCS/PAA) solution became a homogeneous magnetic gel solution. Next step, the gel-forming solution was slowly dropped into 1.0 M sodium hydroxide, which resulted in the formation of spherical hydrogel beads momentarily. The beads were gelled for 1 h and washed ten times with deionized water. Then, wet beads were dipped into glutaraldehyde solution (0.046 mL, 0.12 mmol) and stirred for 12 h at 30 °C to obtain cross-linked magnetic MCS/PAA. After washing thoroughly with deionized water, the cross-linked MCS/PAA beads were filtered and dried at 70 °C for 24 h. The MCS/PAA beads (2 g) were treated with 100 mL of 14% NaOH solution and 1 mL of CS_2_. The mixture was stirred at room temperature for 24 h. Finally, the product was washed thoroughly with distilled water and dried at 70 °C for 24 h.

### 2.3. Analytic Methods

Fourier transform infrared spectroscopy (FTIR) measurements were performed on a Nicolet 6700 spectrometer equipped with a MCT detector. Thermogravimetric analysis (TGA) was undertaken with a NETZSCH STA 449C instrument, and measurements were performed within the temperature ranged from 25 to 700 °C, heating at a rate of 20 °C/min under N_2_ atmosphere. Differential scanning calorimetry (DSC) was performed on a NETZSCH DSC 200 PC unit within the temperature range of −50 to 300 °C, heating at a rate of 10 °C/min under N_2_ atmosphere. Raman spectra were obtained with a laser confocal microscope spectrometer produced by the American Thermoelectric Corporation. The surface morphologies of XMPC were visualized using SEM (SSX-550, Shimadzu, Kyoto, Japan). The X-ray diffraction (XRD) study of the samples was carried out on a Bruker D8 Focus X-ray diffractometer operating at 30 kV and 20 mA with a copper target (l = 1.54 Å) and at a scanning rate of 1° min^−1^. The surface areas were determined by the Brunauer–Emmett–Teller (BET) method (AUTOSORBiQ2, Quan-tachrome, Boynton Beach, FL, USA). Metal ion concentrations were determined by atomic absorption spectroscopy (SSX-550, Shimadzu, Kyoto, Japan).

## 3. Results and Discussion

### 3.1. Preparation and Characterization

**Figure 1:**
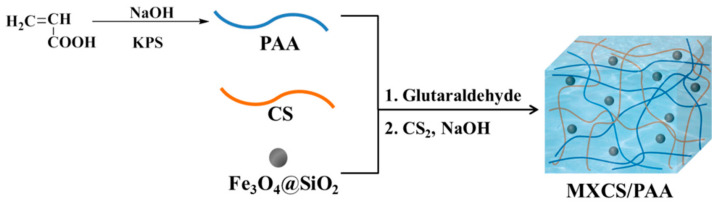
Synthesis route of MXCS/PAA.

MXCS/PAA composite particles were prepared by polymerizing acrylic acid (AA) in an aqueous solution containing chitosan and Fe_3_O_4_@SiO_2_ nanoparticles (Figure 1). Chitosan (CS) was first dissolved in an AA aqueous solution. Fe_3_O_4_@SiO_2_ was then uniformly dispersed in the solution. Thermal decomposition of the initiator KPS generates sulfate anion radicals, which initiates AA copolymerization. Magnetic chitosan/polyacrylic acid (MCS/PAA) was prepared by cross-linking with glutaraldehyde. Finally, MCS/PAA was modified with CS_2_ under alkaline conditions to obtain the xanthate-modified adsorbent MXCS/PAA.

**Figure 2:**
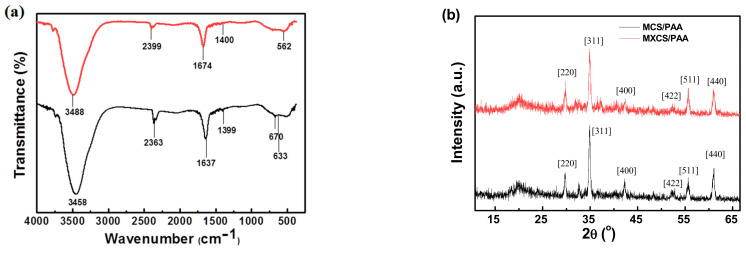
(**a**) FT-IR spectrum, (**b**) XRD spectrum of MCS/PAA and MXCS/PAA.

Fourier-transform infrared (FT-IR) spectra of MCS/PAA and MXCS/PAA are shown in Figure 2a. The peak at 500–600 cm^−1^ is attributed to the vibration of Fe–O in Fe_3_O_4_, indicating that the CS/PAA and Fe_3_O_4_@SiO_2_ magnetic nanoparticles were successfully compounded [20]. The peak at 3488 cm^−1^ is due to the –OH and –NH stretching vibrations of CS/PAA, and that at 2399 cm^−1^ is the C=O vibration of PAA. The peak appearing at 1674 cm^−1^ may be attributed to the C=N bond of the Schiff base formed by the amine group in chitosan and aldehyde group in glutaraldehyde [21].

X-ray diffraction spectra of MCS/PAA and MXCS/PAA are shown in Figure 2b. The characteristic diffraction patterns for Fe_3_O_4_ and SiO_2_ (Figure 2b) have broad peaks at 30.1°, 35.7°, 43.4°, 54.5°, 57.3°, and 62.7°, corresponding to the (2 2 0), (3 1 1), (4 0 0), (4 2 2), (5 1 1), and (4 4 0) planes, respectively [13]. This confirmed that the Fe_3_O_4_@SiO_2_ nanoparticles were successfully composited with the CS/PAA hydrogels. However, by comparing MCS/PAA and MXCS/PAA, it can be observed that the diffraction peak at 20° of MXCS/PAA is weakened. It is the yellowing of chitosan that destroys the hydrogen bonds between chitosans, thereby reducing the hydrogen bonding ability of –OH and –NH_2_, Thus, the ordered structure of the existing chitosan was destroyed to form a certain amorphous structure.

**Figure 3:**
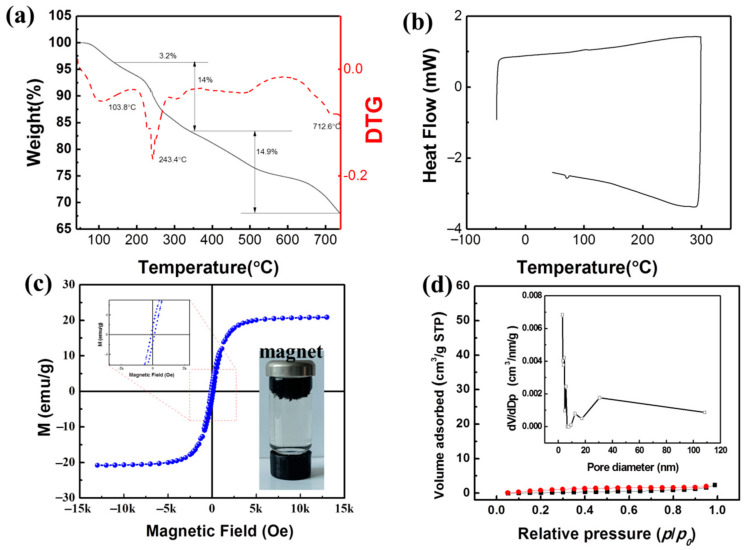
(**a**) TG and (**b**) DSC curves of MXCS/PAA; (**c**) magnetization curves of MXCS/PAA; (**d**) N_2_ adsorption–desorption isotherm of MXCS/PAA. Inset: BJH pore size distribution of MXCS/PAA.

Thermogravimetric (TG) and differential scanning calorimetry (DSC) analysis of MXCS/PAA was undertaken. Figure 3a shows that a slight mass loss occurred below 140 °C, corresponding to the evaporation of absorbed and coordinated water in the material. The most significant mass loss occurred between 140 and 350 °C, which is due to decomposition of the MXCS/PAA skeleton. The final mass loss is attributed to charring [22]. In addition, the TG analysis shows that the as-synthesized magnetic hydrogels are thermally stable. The respective glass transitions of CS and PAA were not observed in the differential scanning calorimetry curve (Figure 3b), which indicated successful formation of the new material [23].

Figure 3c shows the magnetization curve of MXCS/PAA and its magnetic response behavior. The S-curve is symmetrical about the origin, with no hysteresis, remanence, or coercivity, indicating that the material is superparamagnetic. The saturation magnetization was 20.67 emu g^−1^, which is sufficient for magnetic separation in the solution. The decrease in magnetization is due to the fact that the silica or/and chitosan layer can shield the Fe_3_O_4_ core, thereby reducing the magnetic properties of the composite. However, the as-prepared adsorbents can still be easily and quickly separated by an external magnetic field without complicated separation procedures. Figure 3d shows the N_2_ adsorption–desorption isotherm of MXCS/PAA, and the inset shows the corresponding Barrett–Joyner–Halenda pore size distribution. The MXCS/PAA had a surface area of 1.24 m^2^ g^−1^, a total pore volume of 0.004 cm^3^ g^−1^, and an average pore size of 4.9 nm. Importantly, the pore size of the MXCS/PAA composites falls in the mesoporous range (2–50 nm), which is favorable for adsorption. The isotherms are characteristic of type IV adsorption–desorption behavior, showing the typical hysteresis of a mesoporous structure [24].

**Figure 4:**
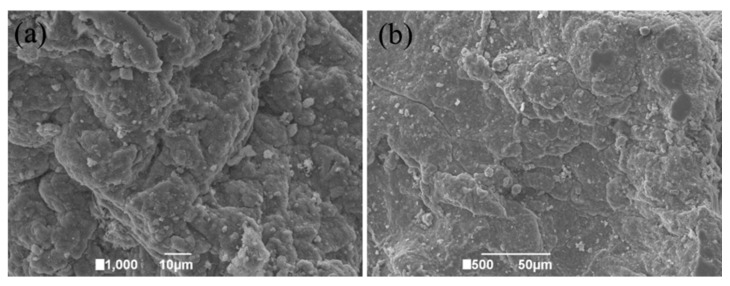
SEM image of MXCS/PAA at (**a**) 1000× and (**b**) 500× magnification.

The MXCS/PAA microstructure was characterized by scanning electron microscopy (SEM), as shown in Figure 4. The MXCS/PAA had a loose structure with interconnected pores, which provided more adsorption sites on the surface and within the adsorbent, and thereby enhanced the adsorption capacity. The surface unevenness and groove shape may be caused by the entry of modifying groups that destroy the original ordered structure.

To investigate the effect of pH on the practical application stability of chitosan-based hydrogels, the MXCS/PAA was tested in deionized water and acidic conditions, for which the swelling ratios were 4.6 and 5.2, respectively. The swelling ratio increased significantly under the primary medium condition, reaching 10.3, probably because the carboxylic-acid functional group of PAA and NaOH increased its swelling ratio. Related data are shown in Table S1.

### 3.2. Evaluation of Adsorption and Desorption Performance

**Figure 5:**
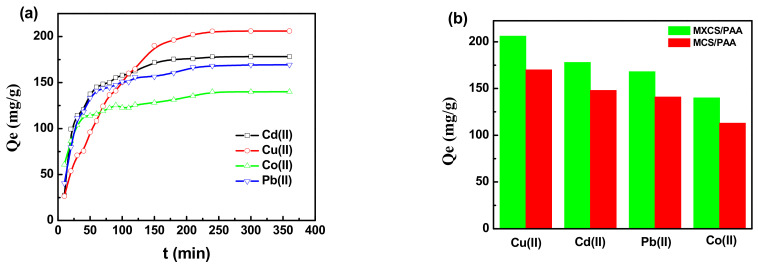
(**a**) Effect of contact time on the adsorption capacity of MXCS/PAA hydrogels at 303 K and 1 g/L, (**b**) adsorption performance of MXCS/PAA and MCS/PAA under optimal conditions.

Figure 5 shows the time-dependent curves of the adsorption capacity of MXCS/PAA for Cu(II), Cd(II), Pb(II), and Co(II) ions from aqueous solution. The adsorption and desorption experiments are shown in the Supplementary Material. In the initial stage of adsorption, the fast adsorption rate is due to the high concentration of heavy metal ions in the solution and many available adsorption groups on the hydrogel [25]. The adsorption capacities reached saturation at about 240 min, at values for Cu(II), Cd(II), Pb(II), and Co(II) ions of 206, 178, 168, and 140 mg/g, respectively. The corresponding adsorption capacities of unmodified MCS/PAA under the same conditions were 170, 148, 141, and 113 mg/g, respectively. The xanthate-modified chitosan-based hydrogel effectively improved the interaction between chitosan and heavy metals in the solution, thereby increasing the adsorption capacity. Related adsorbents were compared with the current adsorbent (MXCS/PAA), and the results are summarized in Table 1.

**Figure 6:**
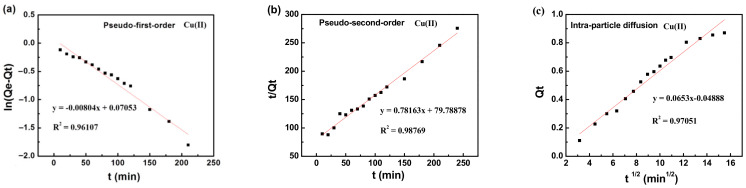
Adsorption kinetic for Cu(II) ions on MXCS/PAA, (**a**) pseudo-first-order kinetic model, (**b**) pseudo-second-order kinetic model, and (**c**) intra-particle diffusion model.

**Figure 7:**
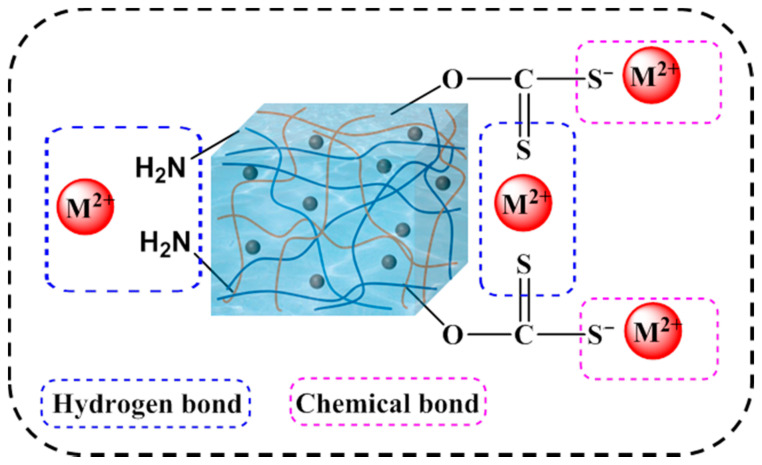
Mechanism of removal of heavy metal ions by magnetic hydrogels.

To further study the adsorption behavior of MXCS/PAA for heavy metal ions, the pseudo-first-order, pseudo-second-order, and intra-particle diffusion kinetic models were fitted to the experimental data. The kinetic models are shown in the Supplementary Material, and the corresponding data are shown in Figure 6 and Table 2. The pseudo-second-order kinetic equation described the adsorption mechanism of the magnetic hydrogels most accurately (R^2^ > 0.99), indicating that adsorption of heavy metal ions by the magnetic hydrogels is a chemisorption process; therefore, adsorption may be accomplished by chelation or electrostatic attraction. Two binding sites are generally required for the adsorption of divalent metal ions. Based on the above results, a mechanism for heavy metal ion removal is proposed, as shown in Figure 7, where M represents the various heavy metals (Pb, Cu, Co, and Cd), the blue dashed boxes are chemical bonds, and the red dashed boxes are hydrogen bonds.

**Figure 8:**
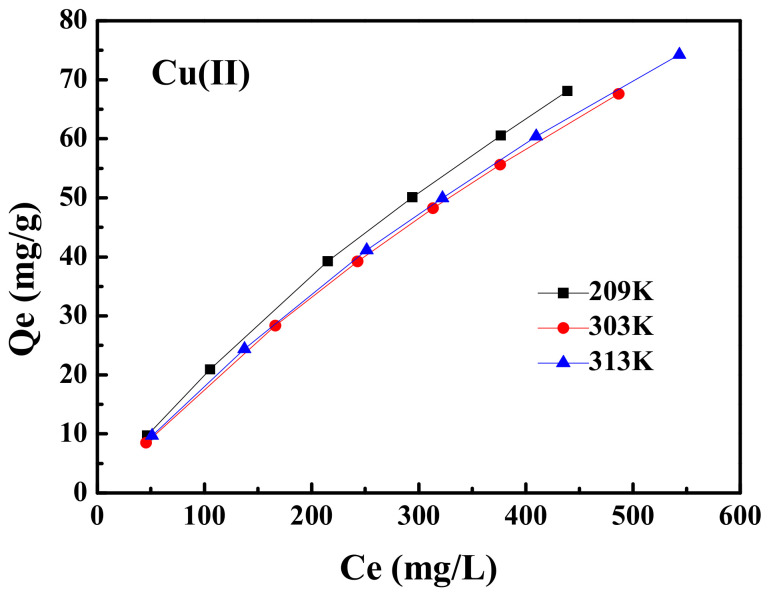
Effect of temperature on the adsorption.

**Figure 9:**
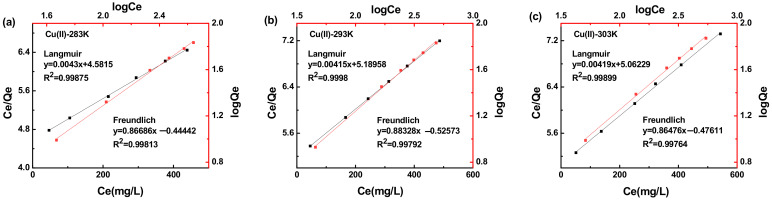
Adsorption isotherms for Cu(II) ions on MXCS/PAA at 283 K (**a**), 293 K (**b**), and 303 K (**c**).

Figure 8 shows the effect of temperature on the adsorption capacity of MXCS/PAA for Cu(II) ions. As the concentration of metal ions increased, the adsorption groups of the magnetic hydrogel were more fully contacted with the metal ions, thereby increasing the adsorption capacity. Fitting of the adsorption isotherm data using the Freundlich and Langmuir models is shown in Figure 9, and the relevant data are summarized in Table 3. The correlation coefficient for the Langmuir model was higher than that of the Freundlich model, which suggests that the active sites were uniformly distributed on the surface during the adsorption process, and adsorption of Cu(II) occurred in single-layer adsorption mode [45,46].

To understand the thermodynamic properties of the adsorption, the standard Gibbs free energy change (Δ*G*), standard enthalpy change (Δ*H*), and standard entropy change (Δ*S*) as functions of temperature were determined using the equations shown in the Supplementary Material. The thermodynamic parameters are presented in Table 4. Δ*H* for the adsorption of Cu(II) was 1.22 kJ/mol. The positive value of Δ*H* indicates that the process is endothermic [47]. The value of Δ*S* > 0 indicates an increase in disorder at the solid–liquid interface during the adsorption process [48].

### 3.3. Desorption and Regeneration

**Figure 10:**
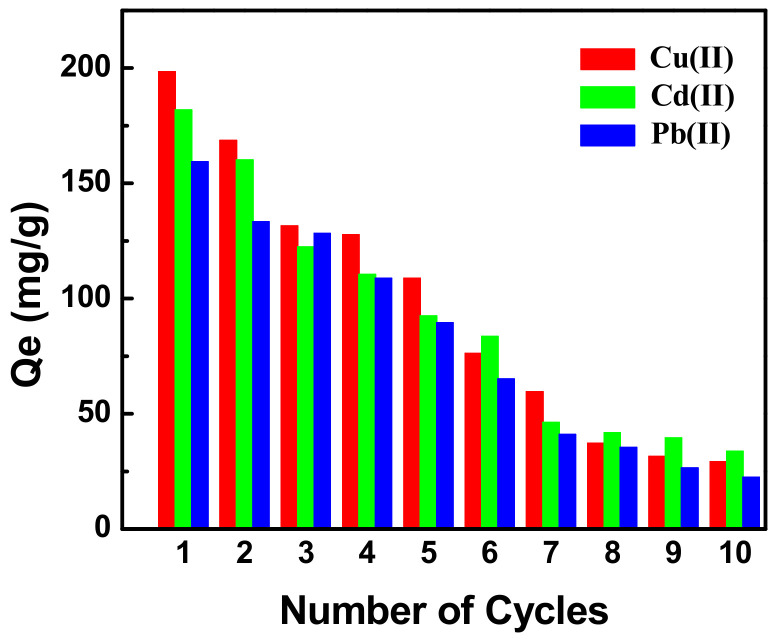
The adsorption capacity of MXCS/PAA on metal ions during regeneration cycles.

Figure 10 shows the desorption kinetics and adsorption–desorption cycles obtained from desorption regeneration experiments using MXCS/PAA. The adsorption capacity of the adsorbent decreased slightly after five consecutive adsorption–desorption cycles, and the adsorption capacity for Cu(II) decreased from 198 to 109 mg g^−1^. The use of hydrochloric acid as an eluent may have destroyed the structure of the magnetic hydrogel, causing a decrease in the desorption rate, which in turn affected the overall adsorption performance of MXCS/PAA. However, the adsorption capacity still exceeded 50% of its initial value after five cycles of adsorption and desorption, indicating that MXCS/PAA has good regeneration performance.

## 4. Conclusions

MXCS/PAA hydrogels were prepared by polymerizing acrylic acid in an aqueous solution of chitosan and Fe_3_O_4_@SiO_2_ nanoparticles. The magnetic hydrogel exhibited good thermal stability and had an average pore size of 4.9 nm, indicating that it is a mesoporous material. Its saturation magnetization was 32.77 emu g^−1^, and it exhibited superparamagnetic properties and good magnetic response characteristics. The xanthate-modified chitosan significantly improved adsorption performance, and MXCS/PAA showed high removal rates for Cu(II), Cd(II), Pb(II), and Co(II). The adsorption process followed a pseudo-second-order kinetic model. The magnetic hydrogel showed good regeneration ability in cyclic adsorption–desorption experiments, retaining 50% of its initial adsorption capacity after five cycles. This study provides some insights into factors influencing the design of adsorbents with good performance and easy recovery for uptake of heavy metal ions. Magnetic hydrogel has good application prospects in water treatment. Its excellent selective adsorption greatly improves the adsorption performance of the material, and it can be specially treated for wastewater enriched with different substances. In addition, the magnetic hydrogel adsorption material is easy to recycle and has a high recycling rate. These advantages can reduce wastewater treatment costs and improve economic efficiency.

## Figures and Tables

**Table 1:** Adsorption of heavy metal from wastewater using magnetic adsorbents.

Adsorbent	Qm (mg/g)													Conditions		Ref.
	Cu^2+^	Pb^2+^	Cd^2+^	Zn^2+^	Co^2+^	Fe^2+^	Ni^2+^	Cr^6+^	Ag^2+^	Hg^2+^	Cr^3+^	As^3+^	As^5+^	pH	T (°C)	
MMWCNT	38.9	-	-	-	-	-	-	-	-	-	-	-	-	9.0	25	[26]
MNPs	76.9	188.7	107.5	51.3	27.7	-	-	-	-	-	-	-	-	6.0	25	[27]
MNR	79.1	112.9	88.4	107.3	-	127.0	95.4	-	-	-	-	-	-	5.5	25	[28]
Fe_3_O_4_/G	-	-	-	-	-	-	-	78.5	-	-	-	-	-	-	25	[29]
3 MPA@Fe_3_O_4_ MNP	-	-	-	-	-	-	42.0	-	-	-	-	-	-	6.0	30	[20]
F-MC	-	-	-	-	-	-	-	1423	-	-	-	-	-	1.0	25	[30]
Magnetite–Dowex 50WX4	416	380	398	-	-	-	384	400	-	-	-	-	-	5.5–7.0	25	[31]
FeNi_3_/TiO_2_	-	-	-	-	-	-	-	398	-	-	-	-	-	3.0	25	[32]
Magnetic reduced graphene oxide-cobalt oxide	-	-	-	-	-	-	-	384	-	-	-	-	-	3.0	25	[33]
FeS-coated iron	-	-	-	-	-	-	-	69.7	-	-	-	-	-	5.0	25	[34]
MNPLB	-	-	-	-	-	-	-	434.8	-	-	-	-	-	2.1	35	[35]
CaO/Fe_3_O_4_	-	227.3	-	-	217.4	-	-	-	-	-	-	-	-	6.0	25	[36]
MCB	122	-	-	-	-	-	-	81	107	306	63	-	-	4.0	30	[37]
Functionalized magnetic microsphere NiFe_2_O_4_	20.2	-	16.6	15.6	-	-	-	-	-	-	16.8	-	-	5.0	25	[38]
MNP-PTMT	-	533.2	216.6	-	-	-	-	-	-	603.2	-	-	-	7.0	25	[22]
CCM	143.3	-	-	-	-	-	-	-	-	-	-	-	-	6.0	25	[39]
MNP-PN-TN	-	-	-	-	-	-	-	35.0	-	-	-	-	-	7.0	45	[40]
MGO-IL	-	-	-	-	-	-	-	-	-	-	-	160.7	104.1	2.0	45	[41]
Magnetic layered double oxide/carbon	192.7	359.7	386.1	-	-	-	-	-	-	-	-	-	-	6.0	25	[42]
MGO	62.9	200.0	-	63.7	-	-	51.0	-	-	-	24.3	-	-	6.0–8.0	25	[43]
Fe_3_O_4_@SiO_2_@CS-P	212.8	-	-	-	-	-	-	-	-	-	-	-	-	6.0	25	[13]
XMPC	100	67	307	-	-	-	-	-	-	-	-	-	-	5.5	30	[14]
MCSB	124.5	-	-	-	-	-	-	-	-	-	-	-	-	5.0	25	[44]
MXCS/PAA	206	168	178		140									5.5	30	This work

**Table 2:** Dynamic parameters for the adsorption of metal ions on MXCS/PAA.

Metal Ions	Pseudo-First Order		Pseudo-Second Order		Intra-Particle Diffusion	
	K_1_ (min^−1^)	R^2^	K_2_ (mg/g min)	R^2^	K_i_ (mg/g min^1/2^)	R^2^
Cu(II)	0.00804	0.96107	0.016520	0.98769	0.0653	0.97051
Cd(II)	0.00878	0.97324	0.066478	0.99924	0.029	0.88909
Co(II)	0.00496	0.89497	0.121432	0.99783	0.0205	0.77431
Pb(II)	0.00756	0.92239	0.027117	0.99623	0.03539	0.75937

**Table 3:** Adsorption isotherm results for Cu(II) ions on MXCS/PAA.

T (K)	Langmuir			Freundlich		
	Q_m_ (mg/g)	K_L_ (L/mg)	R^2^	K_F_ (mg/g)	b_F_	R^2^
283	232.8045	0.000938	0.9988	0.3594	0.8669	0.9981
293	241.2348	0.000799	0.9998	0.2980	0.8833	0.9979
303	238.5657	0.000828	0.9990	0.3341	0.8648	0.9976

**Table 4:** Thermodynamic parameters for the adsorption of Cu(II) ions on MXCS/PAA.

T (K)	K_0_	Δ*G* (KJ·mol^−1^)	Δ*H* (KJ·mol^−1^)	Δ*S* (J·mol^−1^·k^−1^)
283	6.3789	−4.5139		
293	5.8974	−4.3227	1.2221	19.4635
303	6.6160	−4.6028		

## Data Availability

Not applicable.

## Supplementary Materials

The following supporting information can be downloaded at: https://www.mdpi.com/article/10.3390/ijerph191711123/s1. Table S1: Swelling ratio of the MXCS/PAA. Experimental method, adsorption and desorption experiments, swelling experiments, adsorption studies, adsorption kinetics, adsorption isotherms, and absorption thermodynamics.
