# Supplementary material for: Characterization and Mechanistic Study of Heavy Metal Adsorption by Facile Synthesized Magnetic Xanthate-Modified Chitosan/Polyacrylic Acid Hydrogels
Source: Int J Environ Res Public Health. 2022 Sep 5;19(17):11123. doi: 10.3390/ijerph191711123 (PMC9517823; doi:10.3390/ijerph191711123)
Supplement: Supplementary file 1 [file ijerph-19-11123-s001.zip › ijerph-1861242-supplementary.pdf]

Supplementary Materials

Characterization and Mechanistic Study of Heavy Metal Adsorption by Facile Synthesized Magnetic Xanthate-Modified Chitosan/Polyacrylic Acid Hydrogels

Liming Dong [1], Chengyang Shan [1], Yuan Liu [1], Hua Sun [1], Bing Yao [1], Guizhen Gong [1], Xiaodong Jin [2,*] and Shifan Wang [1,*]

[1] School of Material and Chemistry Engineering, Xuzhou University of Technology, Xuzhou 221018, China
[2] Department of Forensic Science and Technology, Jiangsu Police Institute, Nanjing 210031, China
* Correspondence: jinxiaodong@jspi.edu.cn (X.J.); shifanwang@xzit.edu.cn (S.W.)

1. Experimental

1.1. Materials

Chitosan, AA were purchased from Sinopharm Chemical Reagent Co. Ltd., other reagents and chemicals were purchased from Aldrich, Acros and Fisher Scientific unless otherwise noted and used without further purification. All other chemicals were commercially available analytical-grade reagents. All solutions were prepared with deionized water.

1.2. Preparation of MXCS/PAA

First, prepare sodium polyacrylate, add 2.2 mL of acrylic acid solution to 5mL of deionized water, drop 3.2mL of 40% NaOH solution, and stir evenly to make the acrylic acid completely mixed to obtain sodium polyacrylate. Take 3 g of chitosan into a three-necked flask, add 2% acetic acid solution and mechanically stir to dissolve it. After being completely dissolved, add the previously prepared sodium acrylate, then add the initiator 0.08 g $K_2S_2O_8$, $Fe_3O_4@SiO_2$ (1.2 g) was added into the mixture. Subsequently, the composite gel-forming solution was stirred continuously

for 3 h at 30 °C until the $Fe_3O_4@SiO_2$/CS/PAA (MCS/PAA) solution became homogeneous magnetic gel solution. Next step, the gel-forming solution was slowly dropped into 1.0 M sodium hydroxide, which resulted in the formation of spherical hydrogel beads momentarily. The beads were gelled for 1 h and washed ten times with deionized water. Then, wet beads were dipped into glutaraldehyde solution (0.046 mL, 0.12 mmol) and stirred for 12 h at 30 °C to obtain cross-linked magnetic MCS/PAA. After washing thoroughly with deionized water, the cross-linked MCS/PAA beads were filtered and dried at 70 °C for 24 h. The MCS/PAA beads (2 g) were treated with 100 mL of 14% NaOH solution and 1 mL of CS_2. The mixture was stirred at room temperature for 24 h. Finally, the product was washed thoroughly with distilled water and dried at 70 °C for 24 h.

1.3. Analytic Methods

Fourier transform infrared spectroscopy (FTIR) measurements were performed on a Nicolet 6700 spectrometer equipped with a MCT detector. Thermogravimetric analysis (TGA) was undertaken with a NETZSCH STA 449C instrument, and measurements were performed within the temperature ranged from 25 to 700°C, heating at a rate of 20 °C/min under N_2 atmosphere. Differential scanning calorimetry (DSC) was performed on a NETZSCH DSC 200 PC unit within the temperature range of -50 to 300°C, heating at a rate of 10°C/min under N_2 atmosphere. Raman spectra were obtained with a laser confocal microscope spectrometer produced by American Thermoelectric Corporation. The surface morphologies of XMPC were visualized using SEM (SSX-550, Shimadzu, Japan). The X-ray diffraction

(XRD) study of the samples were carried out on a Bruker D8 Focus X-ray diffractometer operating at 30 kV and 20 mA with a copper target (l = 1.54 Å) and at a scanning rate of 1° min^{-1}.The surface areas were determined by the Brunauer–Emmett–Teller (BET) method (AUTOSORBiQ2, Quan- tachrome, USA). Metal ion concentrations were determined by atomic absorption spectroscopy (SSX-550, Shimadzu, Japan).

1.4. Adsorption and Desorption Experiments

Adsorption To evaluate the adsorption capacity of MXCS/PAA for metal ions, adsorption experiments were carried out by adding 0.1 g MXCS/PAA and 100 mL metal ions solution to 500 mL beaker. The solution was mechanically stirred at 303 K, and samples of 0.5 mL were collected at different time intervals for the analysis of metal ions concentration.

Desorption After adsorption equilibrium, the saturated metal ions -loaded adsorbent was magnetically collected by a powerful magnet. Collected XMPC was added to 100 mL 0.1 M HCl solution, and the mixture was mechanically stirred for 2 h at 303K. At the designed time intervals, a sampling of 0.5 mL solution was collected to determine the metal ions concentration. Nine consecutive cycles of adsorption–desorption runs were carried out. After each cycle run, XMPC was magnetically separated and washed thoroughly with deionized water. All tests were conducted in duplicate.

1.5. Swelling Experiments

To evaluate the durability of MXCS/PAA, deionized water (pH = 7), acidic (pH = 3), and basic media (pH = 11) were used for the swelling test. One gram sample

immersed in deionized water, acidic, and basic media for 10 days, respectively. Then the swollen samples were weighted immediately after removing excess water. The swelling ratio (Sw) is calculated as:

$$Sw = (Ws - Wd)/W d \qquad (S1)$$

where Wd and Ws are the masses before and after immersed for 10 days, respectively.

2. Results and Discussion

2.1. Adsorption studies

The adsorption capacity Q_e (mg/g) of MXCS/PAA toward metal ions at equilibrium was calculated as following Equation (S1):

$$Q_e = \frac{(C_0 - C_e) \times V}{m} \qquad (S2)$$

where C_0 and C_e are the initial and equilibrium concentrations (mg/L) of metal ions in solution, respectively. V (L) is the volume of solution and m (g) is the mass of XMPC.

2.2. Adsorption Kinetics

Pseudo-first-order model:

$$\lg(Q_e - Q_t) = \log Q_e - k_1 t \qquad (S3)$$

Pseudo-second-order model:

$$\frac{t}{Q_t} = \frac{1}{k_2 Q_e^2} + \frac{1}{Q_e} t \qquad (S4)$$

Intraparticle diffusion model

$$Q_t = k_3 t^{1/2} + C \qquad (S5)$$

Where k_1 (1/min) and k_2 [g/(mg·min)] are the rate constants for first-order and second-order models, respectively; k_3 [mg/(g·min$^{1/2}$)] is a constant related to the diffusion coefficient in intraparticle diffusion model; C is the intercept for the intraparticle diffusion model; Q_e is the fitted adsorption value (mg/g) at equilibrium, and Q_t is the experimental value (mg/g) at set time t (min), respectively.

2.3. Adsorption Isotherms

Langmuir model

$$\frac{C_e}{Q_e}=\frac{C_e}{Q_m}+\frac{1}{K_l Q_m} \quad \text{(S6)}$$

Freundlich model

$$logQ_e=b_F logC_e+logK_F \quad \text{(S7)}$$

Where Q_m is the maximum adsorption capacity (mg/g), C_e is the final equilibrium mercury concentration (mg/L), K_L is the Langmuir constant (L/mg) related to the adsorption strength. K_F is the Freundlich constant related to the adsorption strength (mg/g) (L/mg), Langmuir isotherm describes a monolayer adsorption which takes place at homogeneous sites within the adsorbent where all the adsorption sites are energetically identical. Freundlich isotherm expresses adsorption at multilayer and on the energetically heterogeneous surface and active sites.

2.4. Absorption Thermodynamics

$$\Delta G^o = -RTlnK_0 \quad \text{(S8)}$$

$$lnK_0 = \frac{\Delta S^o}{R} - \frac{\Delta H^o}{RT} \quad \text{(S9)}$$

Where K_0 is distribution coefficient, , R is the gas constant (8.314 J / (mol·K)), T is the

temperature (K).ΔG_0 is the standard Gibbs free energy change, ΔH0 is the standard enthalpy change (J/mol), and ΔS_0 is the standard entropy change (J/(mol·K)). K_0 was obtained from the intercept of plotting ln(qe/Ce) versus Ce at three different temperatures by extrapolating Ce to zero. ΔH_0 and ΔS_0 were obtained from the slope and intercept in the curve of $\ln K_0$ versus T^{-1}.

2.5. Selective Adsorption Behaviors of Heavy Metals

The selectivity of XMPC for metal ions over other metal ions can be evaluated using the selectivity coefficient (Ks). It is an important parameter to express the removal selectivity of the adsorbent toward objective metal ions in a complex components solution, which can be derived by the following Equation (S10):

$$K_s = \frac{K_d(T)}{K_d(I)} \qquad \text{(S10)}$$

where Kd is the distribution coefficient of metal ions and was calculated by Equation (S11):

$$K_d = \frac{Q_e}{C_e} \qquad \text{(S11)}$$

Table S1. Swelling ratio of XMPC.

pH	W_d (g)	W_S (g)	S_w
3	0.20	1.24	5.2
7	0.20	1.12	4.6
11	0.20	2.25	10.25
